# Clinical Outcomes From Cultivated Allogenic Stem Cells vs. Oral Mucosa Epithelial Transplants in Total Bilateral Stem Cells Deficiency

**DOI:** 10.3389/fmed.2020.00043

**Published:** 2020-02-18

**Authors:** Ovidiu Samoila, Diana Gocan

**Affiliations:** Department of Ophthalmology, “Iuliu Haţieganu” University of Medicine and Pharmacy, Cluj-Napoca, Romania

**Keywords:** stem cells, cornea, cultivation techniques, allogenic, limbal transplant, oral mucosa

## Abstract

Total bilateral limbal stem cell deficiency results from various pathologies, from burns (either chemical or physical) to Sjogren Syndrome, aniridia or ocular cicatricial pemphigoid. After the loss of stem cells, normal corneal epithelium is replaced by a more opaque and vascularized conjunctival epithelium, causing loss of vision. After 1997, cultivation techniques for limbal stem cells became possible. In parallel, cultivation techniques for oral mucosa epithelial cells were also available. The aim of our review was to summarize the clinical outcomes following allogenic cultured limbal stem cell transplant (allogenic CLET), and on the other hand, oral mucosa derived epithelium transplant (cultivated oral mucosa epithelial transplant—COMET or cultivated autologous oral mucosal epithelial cell sheet—CAOMECS), in the case of total bilateral limbal stem cell loss. Thirty studies matching the inclusion criteria were found. The clinical improvement in both methods was reported similar, with percentages higher than 50% of the treated cases. However, the comparison between studies was difficult to achieve due to the lack of a universal and objective grading tool for assessing post-operative results. The definition of clinical improvement was problematic, because success was defined differently, depending on the study. Moreover, some of the studies followed both autologous and allogenic CLET, but described the results together, for both procedures, and therefore it was impossible to analyze them separately. COMET presented some advantages compared to CLET. By using autologous cells, there was no risk of immune activation and no immunosuppression was needed. COMET, however, might be associated with increased risk of persistent epithelial defects and graft failure, compared with allogenic CLET.

## Introduction

The reservoir of corneal epithelial stem cells, a quiescent cell population with proliferative capacity, is located in a niche in the palisades of Vogt, deep in the structure of the limbus, in the basal epithelial layer. Their distribution along the limbus is not regular; they are mostly present in the nasal region and in the mid or distal portion of the limbus toward the conjunctiva. In addition to stem cells, niches consist also of non-stem cells, such as limbal stromal fibroblasts, melanocytes and immune cells which have the role of maintaining the stem cells dormant (1, 2). Following an injury of the corneal surface, the limbal epithelial stem cells (LESCs) proliferate, divide, migrate and mature in order to ensure regeneration (3). LESCs are activated into transient amplifying cells, divide and migrate toward the center of the cornea in order to restore the surface. LESC activation is dependent on growth factors, cytokines, extracellular matrix, and integrin receptors (2, 4). Dysfunctional or destroyed LESCs define corneal limbal stem cell deficiency (LSCD). Clinically, LSCD causes loss of corneal transparency and replacement of the corneal epithelium with conjunctival epithelium, scarring, vascularization, and persistent epithelial defect (PED) or recurrent erosions. LSCD may appear following exogenous factors: infections, chemical, or thermal burns, long-term use of topical medication, contact lenses, irradiation, tumors, surgery, and medical conditions, such as Steven–Johnson syndrome, aniridia or ocular pemphigoid (5, 6). LSCD can be unilateral or bilateral, partial or total, according to the extent of deficiency of stem cells. Partial LSCD means that there are regions where the population of stem cells is normal; therefore conjunctivalization occurs only in those regions where cells are insufficient. Total LSCD results in conjunctivalization of the entire cornea (7).

The management of corneal LSCD has changed over the years. The surgical treatment is dependent on the unilaterality/bilaterality of the condition. In 1989, Kenyon and Tseng (8) performed conjunctivolimbal autograft transplantation (CLAU). The tissue was harvested from the healthy or less damaged eye to treat a contralateral severe LSCD. For patients with bilateral LSCD Tsai and Tseng (9) performed keratolimbal allografting (KLAL). KLAL can successfully restore ocular surface stability in about 50% of bilateral ocular surface disease. Clinical outcome in KLAL is worse than in CLAU and the incidence of complications is higher (graft failure, repeated transplantation, glaucoma). Allografts are obtained either from a living related donor or a cadaver. Immunosuppression is needed in order to prevent the rejection of the graft. Immunosuppression is adapted individually, according to the severity of graft dysfunction, ABO blood type of the patient, reactive antigen and repeated failure of the graft (10).

A more recent development for the management of LSCD is the transplantation of cultured stem cells. The cultured limbal stem cell transplant (CLET) starts with a millimeter-size limbal biopsy from an unaffected site if there is one (9, 11). The limbal biopsy contains populations of LESCs, which are cultivated to provide enough cultured cells for transplantation. One of the advantages of CLET is the use of a smaller limbal tissue. The complications are lower than in CLAU, and further biopsies are possible if needed.

The problem of bilateral total loss of LESCs is more difficult to solve. Autologous source of limbal stem cells is not available in this case. Stem cells can be provided then, by donors (allogenic LESCs), or can be found in other parts of the body (different types of stem cells). The success of the cultivated autografts encouraged the clinical use of allografts obtained from cadavers or living donors (allogenic CLET), in the case of total bilateral loss of LESCs. The problem of immunosuppression remains similar to KLAL but it was speculated that there might be a reduced risk of allograft rejection when using *ex vivo* cultivated cells, which could be explained by the absence of antigen-presenting Langerhan's cells (7).

To overcome the problems of allograft rejection, oral mucosal epithelium was also cultivated and transplanted with favorable results (cultivated oral mucosa epithelial transplant—COMET). Studies have shown that oral mucosa epithelial cells require a reduced time for cultivation and they remain non-keratinized for an extended period of time (12). CAOMECS (cultivated autologous oral mucosal epithelial cell sheet) as an alternative to restore the corneal surface is a newer version of COMET and it uses a culture system which is sensitive to temperature. The cultivated cell sheet can be transplanted without additional support or substrate (13, 14).

## Objective

In this review we examined the two procedures usually applied for the treatment of total bilateral stem cells deficiency, COMET/CAOMECS, and allogenic CLET. We compared the outcomes of the two types of surgeries, the clinical and anatomical rates of success, and the complications. To our knowledge, there is only 1 clinical study comparing the 2 procedures, and it was also included in this study (15).

### Literature Search

This review included studies reporting clinical results following allogenic CLET and COMET/CAOMECS in the past 15 years, regardless of the age of patients or etiology of LSCD. We have explored all the published data in the recognized databases like PubMed, Science Direct, and Scopus by using the terms: “stem cell transplantation; cornea; (allogenic or allogeneic)” and “transplant; cornea; oral mucosa.” In the searched period, from 2004 to 2019, we found 30 studies matching criteria, 18 clinical studies for allogenic CLET, with publication date ranging from 2005 to 2019, 11 clinical studies for COMET or CAOMECS, with publication date from 2004 to 2019, and 1 study comparing the two of them, from 2019. Studies comparing clinical results of CLET or COMET to other surgical techniques were also included, but only data referring to allogenic CLET or COMET were taken into consideration.

## Limbal Stem Cells Culturing Techniques

Literature has described two systems for the *ex vivo* cultivation of LESC: the explant culture system and the suspension culture system. Previous research showed that the two culture systems provide similar results regarding stem cells content (16). The explant system used amniotic membrane, for providing growing substrate and for transporting the cultured cells (4). This process can take from 14 to 28 days. Parihar (17) used the explant technique, placing small pieces of tissue, cut from 2 by 2 mm limbal biopsies, onto denuded amniotic membrane. A more detailed view regarding the size of limbal biopsies is provided in Table 1. The suspension culture system used enzymes in order to separate epithelial cells, obtaining a suspension of cells. 1–2 mm long biopsies were trypsinized and introduced into a suspension culture system, establishing multiple cultures. Half of the corneo-scleral rim was used in order to achieve cells suitable for culture. The cells from the suspension were arranged on a culture dish, culture medium was added and then the cells were incubated between 14 and 21 days. After this period of time, when cells were prepared to be transplanted, carriers, such as contact lens, amniotic membrane, paraffin gauze, nylon or collagen shields, were used for transportation of stem cells to the ocular surface (7). Shimazaki et al. (19) used both techniques, explant and suspension.

**Table 1:** The size of limbal and oral mucosa biopsies in allogenic CLET and COMET/CAOMECS studies.

**Surgical procedure**	**Author, year**	**Size of grafted tissue**
*COMET*	Daya et al. (18)	1–2 mm
	Shimazaki et al. (19)	Not mentioned
	Kawashima et al. (20)	1 × 3 mm
	Shortt et al. (21)	2–3 mm
	Pauklin et al. (22)	1 × 2 mm
	Basu et al. (23)	2 × 2 mm
	Prabhasawat et al. (24)	2 × 2/3 × 1 mm
	Qi et al. (25)	Not mentioned
	Shortt et al. (26)	Not mentioned
	Zakaria et al. (27)	Not mentioned
	Ramírez et al. (28)	2 × 2 mm
	Ganger et al. (29)	2 × 2 mm
	Parihar et al. (17)	2 × 2 mm
	Chen et al. (30)	Not mentioned
	Cheng et al. (31)	Not mentioned
	Behaegel et al. (32)	1 × 2 mm
	Borderie et al. (33)	1 mm
	Campbell et al. (34)	Not mentioned
	Wang et al. (15)	Not mentioned
*COMET/CAOMECS*	Nishida et al. (35)	3 × 3 mm
	Chen et al. (36)	6 × 6 mm
	Nakamura et al. (37)	Not mentioned
	Satake et al. (38)	8 mm punch
	Priya et al. (39)	4 × 2 mm
	Burillon et al. (13)	3 × 3 mm
	Sotozono et al. (40)	6 mm
	Kolli et al. (41)	3 mm
	Kocaba (14)	3 × 3 mm
	Dobrowolski et al. (42)	3–5 mm^2^
	Kim et al. (43)	0.8 × 1.5 – 1 × 2 cm^2^
	Wang et al. (15)	4 × 4 mm

The culture medium consisted of: complete growth medium of Dulbecco's modified Eagle's medium, F12, irradiated fetal bovine serum, hydrocortisone, cholera toxin, recombinant human insulin, epidermal growth factor and antibiotics (18, 21–23). Some studies used autologous serum from the donor to make a xeno free transplant (22, 23). Epithelial cells were cultivated on a cryopreserved and partially decellularized amniotic membrane. One study mounted the cells on a non-adherent nylon dressing (18). Cultivation success was defined when a monolayer of cells became confluent (17).

The tissue from a cadaveric donor was obtained within 24 h (22) to 7 days post-mortem (28). One study obtained cadaveric tissue within 2–3 weeks before the planned allogenic CLET procedure (24). Basu et al. (23) used only tissue from living donors, Daya et al. (18), Shimazaki et al. (19), and Pauklin et al. (22) used both sources (living donor and cadaveric). Corneo-scleral rings discarded after corneal graft harvesting procedures were used in order to achieve cells suitable for culture.

Most culture protocols used 3T3 feeder cells in order for the graft to receive proper nutrients, but they also have a role in detoxifying the culture media and providing extracellular matrix proteins (44). Inactivated 3T3 fibroblast cells were previously described to enhance proliferation (7, 45). These feeder cells undergo inactivation using mitomycin C or irradiation. Because of their animal exposure during the culture process, there may be a higher risk of rejection and possible viral infection. Some protocols included human-derived feeder cells layers instead of the 3T3 feeder cells (46), because they were associated with a higher safety profile.

Some authors conducted morphological and immunohistochemical studies in order to determine the cellular morphology after cultivation (45, 46).

## Oral Mucosal Epithelial Cultivation

After the oral cavity was sterilized, a specimen of oral mucosa was excised from the interior buccal mucosal epithelium (35). The tissue harvested varied in size—from 3 by 3 mm (35) to 10 by 20 mm (17). The size of oral mucosa biopsies is detailed in Table 1. Cell isolation and cultivation methods were similar to those described above. Cell suspension techniques were applied in all studies, with one exception (17).

## Surgical Transplantation

### Surgical Technique

Preoperative preparations in most allogenic CLET studies included tissue screening for human immunodeficiency virus 1 and 2, syphilis, hepatitis B and C (21–23, 28, 29). Cheng et al. documented a similar screening protocol but did not include testing for syphilis (31). Authors from two studies also tested the tissue for human T cell leukemia-lymphoma virus (21, 28). One study refers to using a previous protocol where preoperative screening was performed (26). The other CLET studies which we included in this review did not mention screening protocols (20).

The transplantation procedure included a 360° conjunctival peritomy and removal of the modified corneal epithelium. The cultivated limbal stem cells were transferred onto the ocular surface on amniotic membrane, which was secured with sutures in all studies with two exceptions, Zakaria et al. (27), who secured the graft with fibrin glue, and Ganger et al. (29), who used both, either sutures or fibrin glue. In some studies a bandage contact lens was inserted after the transplant (21, 24, 26–29, 31) while in other studies a second amniotic membrane was used as a patch (18, 22, 27, 34). One study mentioned using either bandage contact lens or amniotic membrane (20) whereas one study does not mention the use of either (17) In cases of symblepharon, fibrovascular tissue surrounding rectus muscles was dissected in order to achieve normal ocular movement (31).

In COMET and CAOMECS, the transplantation technique was similar to allogenic CLET. The cultivated oral mucosal epithelial sheets were transferred to the corneal surface after removing the modified epithelium. In most cases, cultured sheets were not secured with sutures (13, 14, 35, 37, 43), however a therapeutic contact lens was used over the transplant. Nonetheless, a few authors reported suturing cultivated sheets to the conjunctiva (36, 38, 42). With patients also suffering from cataract, phacoemulsification and posterior chamber implant were performed simultaneously with COMET (40). Kolli et al. (41) does not mention the exact transplantation technique. Priya et al. (39) additionally used mitomycin C for subconjunctival spaces.

### Post-operative Considerations

Post-operative management mainly included topical antibiotics, immunosuppression and lubricating eye drops and systemic immunosuppression. Post-operative therapy for all allogenic CLET studies included topical antibiotics (ciprofloxacin 0.3%, tobramycin 0.3%, ofloxacin 3%, levofloxacin or chloramphenicol 0.5%) with two exceptions (19, 26). Local immunosuppression was obtained by topical prednisolone acetate 1%, fluorometholone 0.02%, dexamethasone 1%/0.1%, or methylprednisolone 1%. Cyclosporine A drops were included as topical regimen in two studies (19, 31). On the 1 days after surgery, patients were administered oral immunosuppression agents. Systemic immunosuppression was obtained by Cyclosporine A and steroids. One study described using mycophelonate mofetil and azathioprine also (28). In one case, a patient received mycophenolate mofetil, due to the fact that cyclosporine A was not tolerated (22). Autologous 20% serum eye drops were also included in the 1 week post-operative by some authors (18, 22, 24, 27, 31). One study documented inducing ptosis in all patients during the 1 week following CLET (22). In all studies, immunosuppression was slowly tapered within months after the transplant.

A study which analyzed the outcomes of allogenic CLET and DNA analysis of donor, suggested that immunosupression more than 9 months following CLET might be not be needed (18).

Similarly, following COMET/CAOMECS, patients received antibiotics and anti-inflammatory agents [topical or general corticosteroids, or Cyclosporine (37)], in order to keep inflammation to the minimum, and artificial tears (13, 14, 35–38, 40–43). In some studies, autologous serum drops were used also (41, 42, 47).

## Clinical Outcomes

Clinical outcomes following allogenic CLET and COMET/CAOMECS for bilateral limbal stem cell deficiency of different etiologies are summarized in Table 2, in chronological order. There is no common view regarding the assessment of the clinical success. The outcomes of the surgery were mostly evaluated based on clinical and functional findings. Impression cytology or confocal microscopy, as outcome measures, were described by Shortt el al. (21, 26). Impression cytology alone was used by Daya et al. in order to perform DNA analysis (18) and by Prabhasawat et al. (24). Shimazaki et al. (19) used impression cytology in order to confirm conjunctivalization preoperatively and to asses corneal phenotype following surgery. *In vivo* confocal microscopy was used by Ramirez et al. to assess epithelial phenotype of the central cornea 1 year following CLET (28).

**Table 2:** Results following CLET and COMET.

**Surgical procedure**	**Author**	**LSCD causes**	**No of patients**	**Follow-up**	**Success rate**	**Final VA**	**Complications**
*CLET*	Daya et al. (18)	SjS, burns, other^a^	4	12–50 months	NA (improvement of visual acuity and transparency)	Counting fingers to 20/25	0
	Shimazaki et al. (19)	SjS, burns, other^b^	20	6–85 months	70%; Improvement of BCVA, 40%; DNA analysis	>20/2,000 (14 eyes)	10 primary epithelial failure 4 ulcers 3 infections 4 perforations
	Kawashima et al. (20)	Burns, SjS	4	25.1 months	41.7–62.5%; Stable epithelium	Improvement 2 lines, after PK	1 PED^*^
	Shortt et al. (21)	Burns, aniridia, other^c^	7	13 months	71% (85% clinical signs Improvement)	Light perception to 20/125	1 infectious keratitis 1 graft dehiscence 3 PED
	Pauklin et al. (22)	Burns	14	9–73 months	50%, re-saturation of ocular surface integrity	>20/500	NA
	Basu et al. (23)	Burns, SjS, OCP, other^d^	21	12–120 months	71.4%	20/200–20/40	25% PED and stromal melting^**^
	Prabhasawat et al. (24)	Burns, SjS, other^e^	7	6–47 months	85.7%	Hand movement to 20/40	1 infectious keratitis
	Qi et al. (25)	Burns	41	12–24 months	NA	Not mentioned	10 rejections
	Shortt et al. (26)	Aniridia, SjS	17	36 months	Improvement of BCVA−79% at 6 months, 57% at 24–36 months	Improvement, 2 lines	NA
	Zakaria et al. (27)	Burns, aniridia	3	25–48 months	66%	Counting fingers to 20/100	NA
	Ramírez et al. (28)	SjS, burns, aniridia	9	36 months	66.7 %	No light perception-20/20	0
	Ganger et al. (29)	SjS, burns	8	24.7 months	50 and 37.5% increased BCVA by 1 line 12.5% increased BCVA by 2 lines	Improvement, 1–2 lines	NA
	Parihar et al. (17)	Burns, SjS, OCP, allergy	20	12 months	68% stable surface (Shirmer, BUT, fluorescein staining); 76%—increased BCVA, 19 eyes	Improvement, 2 lines	1 perforation 24% PED within first 2 weeks
	Chen et al. (30)	Burns, trauma, SJS	41	10–89 months	32/41 (78.04%)	20/400 (PK, LPK)	NA
	Cheng et al. (31)	Burns	80	12–60 months	50%	>20/400	18.8% PED 8.8% rejection 41.3% entropion
	Behaegel et al. (32)	Aniridia, burns, microftalmia	4	25 months	1/4 total success; 2/4 partial success	Counting fingers to 20/50	NA
	Borderie et al. (33)	Burns, aniridia, surgeries	7	66–101 months	29% (3 years); 0% (5 years)	Decreased BCVA, by 0.7 lines	40 adverse reactions
	Campbell et al. (34)	Burns, aniridia, OCP	13	18 months	5/8 increased VA	Improvement, up to 20/20	NA
	Wang et al. (15)	Burns	41	23.3 months	71,4%	Improvement, 2 lines	3 PED, 4 rejections
*COMET/CAOMECS*	Nishida et al. (35)	SjS, OCP	4	13–15 months	NA (improvement of visual acuity and transparency)	20/300–20/25	0
	Chen et al. (36)	Burns	4	27–35 months	NA (persistence of cells)	20/400–20/40	NA
	Nakamura et al. (37)	Burns, OCP, other^f^	17	55 months	↑ VA 53% at 36 months	Hand movement to 20/40	PED 5–26%
	Satake et al. (38)	SjS, burns, OCP	36	6–54.9 months	stable surface 64.8% at 1 year, 53.1% at 3 years	0–20/30; >20/125 (PK)	22,5% PED 8 stromal melting 2 infectious keratitis 1 herpetic keratitis^***^
	Priya et al. (39)	Burns, SJS	7	1–34 months	3/7 (42%) anatomical; from which 2 with visual improvement (28%)	Light perception to 20/200	2 rejections
	Burillon et al. (13)^#^	Burns, aniridia, other^g^	26	12 months	75% (16 patients)	Hand movement to 020/50	1 perforation 1 rejection
	Sotozono et al. (40)	Burns, SjS, OCP, other^h^	40	6.2–85.6 months	Improved BCVA: 50%—SJS, 42,9%—OCP, 20%—burns	Improvement, at least $\frac{1}{2}$ line	40% PED, 5% stromal melting, 5% slight-mod infection
	Kolli et al. (41)	Burns	2	21–41 months	NA (stable epithelium)	20/200–20/63	NA
	Kocaba (14)^#^	Burns, aniridia, other^i^	23	28 months	Improved BCVA 74%	Improvement, 2.3 lines	1 perforation 1 rejection
	Dobrowolski et al. (42)	Aniridia	13	12–18 months	76.4% stable surface; Improved BCVA 88.2%	Hand movement−20/200	3 graft failures
	Kim et al. (43)	Burns, SjS	8	2–15 months	75%	Improvement > 2 lines	50% PED
	Wang et al. (15)	Burns	32	16.1 months	52.9%	Decreased -improvement 2 lines	9 PED, 5 stromal melting

# CAOMECS.

* *Complications post-PK: 2 endothelial rejections (months 18–24)*.

** *Complications post-PK: 69.2% graft failure, 33.3% endothelial rejection, 11.1% traumatic dehiscence, 22.2% recurrence of LSCD*.

*** *Complications post-PK: 1 perforation, 1 endothelial rejection*.

*SjS, Sjogren Syndrome; OCP, ocular cicatricial pemphigoid; PED, persistent epithelial defect; NA, not available; BCVA, best corrected visual acuity*.

a *Rosacea blepharoconjunctivitis, ectodermal dysplasia*.

b *OCP, keratoconjunctivitis, unknown cause*.

c *Ectodermal dysplasia, Reiger's anomaly*.

d *Allergy, contact lens hypoxia*.

e *Allergy, dry eye syndrome, multiple eye surgeries*.

f *Squamous cell carcinoma, graft vs. host disease*.

g *Contact lens hypoxia, Lyell syndrome, rosacea keratitis, neuroparalytic keratitis, Groenow dystrophy, trachoma, hepatitis C, cystinosis*.

h *Graft vs. host disease, Salzmanns degeneration, radiation keratopathy, drug induced LSCD, idiopathic LSCD*.

i *Contact lens hypoxia, Lyell syndrome, rosacea keratitis, neuroparalytic keratitis, Groenow dystrophy, trachoma, hepatitis C, cystinosis*.

### Allogenic CLET Studies

Daya et al. (18) reported an improvement of LSCD in 7 patients, 70% of the cases. All were severe LSCD, with four quadrants involvement. Mean follow up was 28 months. Parameters followed were vascularization, conjunctivalization, inflammation, epithelial defect, photophobia and pain. The seven patients considered a success also had performed impression cytology with DNA analysis. This revealed no donor DNA present on ocular surface in 5 out of the 7 cases tested between 1 and 7 months post-op. The other two cases had positive donor DNA present at 2 and 7 months, respectively, but not after 9 months. Four cases (40%) also had an improvement in visual acuity. Three of these cases underwent perforating keratoplasty (PK) and supplemental KLAL at different intervals after the main surgery. Three other patients received KLAL together with forms of keratoplasty (PK, DALK, lamellar) and amniotic membrane transplantation. Second procedures were performed to improve vision of for tectonic reasons at 5–40 months after the first surgery. KLAL was supposed to act as a barrier for conjunctivalization. Best visual acuity was 20/25 but the other three cases reached 10/100, 20/100, and 20/80.

Shimazaki et al. (19) reported 62.5% success rate for stem cells provided by living relatives donors and 41.7% for unmatched cadaveric donors. Their study included 27 eyes from 27 patients and the mean follow-up time was 127 weeks. Success in ocular surface reconstruction was defined as having stable epithelium with corneal phenotype on the central cornea with and without peripheral conjunctival invasion. Corneal phenotype was evaluated with impression cytology or slit-lamp examination with fluorescein staining. There were complications reported but the report did not differentiate between autologous and allogenic technique.

Kawashima et al. (20) evaluated cell phenotype of the central cornea post-operative. Slit lamp examination and fluorescein staining showed corneal phenotype after allogenic CLET surgery. Histopathological examination revealed corneal phenotype in 4 of 6 patients. Epithelial cells were positive for K3 and negative for K13, which indicated the presence of normal corneal epithelium. The follow-up period ranged from 5 to 41 months. A decrease of the neovascularization was also recorded. Visual acuity was not significantly improved due to persistent stromal opacification, however, it was improved when additional procedures were performed. One case of persistent epithelial defect was present, but it responded well to topical treatment.

Ramirez et al. (28) studied post-operative results by dividing patients into three categories: burns, inflammations related to autoimmunity and non-inflammatory diseases. Success rate was 66.7% at the 1 year follow-up for patients who underwent allogenic CLET. *In vivo* confocal microscopy performed at the 12 months follow-up was used to determine the presence of corneal phenotype after CLET. Following allogenic CLET, five cases which initially were allocated to having conjunctival-like or mixed epithelial cell phenotype, had improved to corneal phenotype. One patient presented mixed phenotype. No adverse reactions or rejection was recorded.

The study carried out by Ganger et al. (29) mainly focused on results of allogenic transplant in children compared to adults. The mean follow-up time was 24.7 months. Out of the eight patients, five were children (patients under the age of 15) and three were adults. A successful surgery was defined by anatomical and visual acuity improvement. Anatomical success was observed in 50% of the cases and visual acuity was improved by one line in 37.5% of cases and by 2 lines in 12.5% of cases. Nonetheless, comparison of the results between the two groups was not made due to the small amount of patients.

Shortt et al. (21) studied eight cases of allogenic transplant and reported an improvement of clinical signs in 85% of the them. A successful surgery was defined by improvement of the visual acuity, absence of signs of LSCD, corneal phenotype on impression cytology and presence of corneal cells at confocal microscopy. At the 6 months follow-up patients had increased corneal transparency, reduced or absent corneal vascularization and the corneal surface was smooth. Impression cytology and confocal was used to evaluate the presence of corneal morphology. In impression cytology, samples were immunostained with monoclonal antibodies in order to evaluate the phenotype of the cells from the corneal surface. Results were compared preoperative and post-operative. Corneal phenotype was present in 5/7 of the samples. There were only few complications: one graft failure was reported and a secondary transplant was performed. The overall success rate of CLET was 71%.

Pauklin et al. (22) considered that a completely restored ocular surface was the major success of the surgery and it was achieved in 50% of the cases. The mean follow-up time was 28.5 months. Improvement of preoperative and post-operative visual acuity was considered less important, however it was documented in 64.3% of the cases. In eyes which previously suffered a chemical or thermal injury, the visual acuity improved the most. A successful surgery meant a smooth and clear corneal surface, without epithelial defects and without recurrence of conjunctivalization. Failure of surgery was represented by loss of transparency of the corneal surface, superficial vascularization in more than one quadrant and persistent epithelial defects and was documented in 4 eyes (partial success in 4 eyes).

A stable corneal surface was also considered a success by Basu et al. (23) and it was reported in 71.4% of the cases. LSCD was defined as 360° superficial corneal vascularization, diffuse fluorescein staining of the corneal surface with or without persistent epithelial defects, conjunctivalization of the corneal surface and absence of limbal palisades of Vogt. The recurrence of epithelial defects or superficial corneal vascularization was defined as failure of surgery. Measurement of visual acuity and evaluation of possible complications were also studied. Although VA improved in all 28 eyes, failure as defined was documented in 8/28 eyes (28%; 75% of the failures were between 1 and 9 months). Some complications were related to the instability of the corneal surface, whereas others were related to the immunosuppressive treatment. PK was performed in 13 eyes 14 months (average duration between allogenic CLET and PK was 12–22 months) after CLET if the VA was worse than 20/60 and attributed to corneal stromal scarring. Authors did not mention the follow-up time post-PK but the mean follow-up time after CLET was 4.8 years. Histology performed on the excised cornea showed no sign of amniotic membrane or goblet cells and epithelial cells expressed cornea specific marker K12.

While a stable corneal surface was the main outcome measured by most authors, Prabhasawat et al. (24) also included histological outcomes to determine success after allogenic CLET. A good histological outcome was characterized by the absence of goblet cells in the central cornea. Interestingly, the success rate was higher in the allogenic group. The reported success rate was 85.7% after allogenic CLET vs. 66.7% after autologous CLET. However, lid abnormalities, more common in the autograft group, could explain the results. There was one graft failure due to infectious keratitis.

Zakaria et al. (27) assessed both anatomical and functional success after CLET. Improvement of pain, photophobia and visual acuity was a functional success. They reported 2 out of 3 successful cases following allogenic CLET. There was one failed case, which presented a lack of epithelialization of the corneal surface and conjunctivalization.

Parihar et al. (17) described improvement of visual acuity after CLET and lack of conjunctivalization in 12 out of 20 patients (60%). At the 1 year follow-up, in 14 out of 24 eyes (58.33%) there was no conjunctivalization noted. Ocular surface stability was evaluated by Schirmer test, BUT and fluorescein staining. However, persistent epithelial defects were recorded in 6 cases within 2 weeks after surgery. One case of perforation following allogenic CLET and some cases of adverse reactions due to immunosuppressive therapy were reported.

Cheng et al. (31) evaluated primary and secondary outcomes of CLET in 80 cases of symblepharon, after a follow-up of 12–60 months. Complete success was represented by the absence of scars or ocular motility restriction and the presence of a deep conjunctival fornix. Secondary outcomes were evaluated based on: visual acuity at follow-up compared to visual acuity preoperative, presence of complications, risk factors for the recurrence of symblepharon and surgery for recurrent symblepharon. The success of the surgery was associated with the severity and the inflammation of the symblepharon. The complete success rate was 50% after first surgery (40 eyes), partial success was 31.3%, and failure was 18.8% (15 eyes). Corneal condition (diminished conjunctivalization) improved in 43 eyes (53.8%). The cause of the symblepharon had an influence on the outcome of the surgery. In patients with symblepharon following thermal injuries, success was achieved in 31 out of 44 cases. The success rate was higher in cases of symblepharon following chemical injuries, 34 out of 36 cases−94.4%. Recurrent symblepharon was documented in 50% of cases during the first 3 months post-operatively. Eyes with thermal injuries recorded a more rapid recurrence. Complications, such as immune rejection or recurrent epithelial defects were recorded. Nonetheless, a grading system would be required for a better evaluation of post-operative outcomes. In 2014, Shortt et al. (26) tried to design a grading system for a better and objective assessment of results. Clinical signs related to LSCD that were assessed by previous studies were included in the grading system. The most relevant four clinical signs were included: corneal epithelial haze, epithelial defect, irregularity of the epithelium and superficial vascularization. Each of these parameters was attributed with grades from 0 (normal) to 3 (severe). Photographs of the cornea were also included, which were taken according to specific parameters (high magnification ×16 of the central cornea, under blue cobalt illumination after one drop of fluorescein 2% was instilled). Based on the grades given to the clinical signs and photographs, the severity (which ranged from 0 to 12) was analyzed. This grading system is titled The Clinical Outcome Assessment in Surgical Trials of Limbal stem cell deficiency—COASTL. With the use of COASTL grading system, each case was graded a global score, based on clinical parameters, which allowed a better and objective evaluation. However, to our knowledge this grading system was not used in other studies. This study included cases of aniridia and Stevens Johnson syndrome. Improvement of visual acuity was documented in 79% of the eyes at the 6 months follow-up, 71% of eyes at the 12 months follow-up and 64% of eyes at the 18 months follow-up. In patients with aniridia, improvement of the global score was noted 12 months after CLET. LSCD associated with Stevens Johnson syndrome recorded an improvement 6 months after surgery, and then between 6 and 18 months post-operatively signs of LSCD were observed. After 18 months, a stabilization and improvement was again documented.

Borderie et al. (33) aimed to compare outcomes following cultured LESCs transplantation to limbal tissue transplantation. They included 30 eyes with LSCD stage III, which is characterized by vascularization of the entire cornea, irregular epithelium, staining of the entire limbus and central cornea. Seven patients underwent allogenic CLET and 8 underwent allogenic limbal transplantation. The survival of the graft at the 3-years mark was 29% for allogenic CLET, 50% for allogenic limbal transplantation and 0%, respectively, 33% at 5 years following surgery. A reduction in visual acuity was recorded (0.7 lines for allogenic CLET and 1.9 lines for allogenic limbal tissue transplantation).

Behaegel et al. (32) followed four patients which underwent allogenic CLET for a mean period of 2.1 years to establish the short and long term results of surgery. Long term results showed that all cases could either be categorized as partial success or failure.

Campbell et al. (34) conducted a randomized clinical study and distributed the patients into two groups: one which received allogenic corneal epithelial stem cells on amniotic membrane and one which received only amniotic membrane. Both groups received immunosuppression. Sixteen patients were included but only 13 remained at the end of the follow-up period. The mean follow-up was 32 months. Improvement in visual acuity was recorded in both groups; however, in these cases cataract surgery was also performed. Ocular surface was more stable and received a lower score in the group of allogenic CLET. The main purpose of the study was to establish if there is a long-standing and superior outcome post-transplant and concluded that allogenic CLET is feasible and safe procedure in severe bilateral LSCD.

The characteristics of immune rejection following allogenic CLET was studied by Qi et al. (25). They included 41 patients in their study and the recorded immune rejection rate was 23.8% (10 eyes, 9 patients). Mean duration between surgery and immune rejection was 3.1 months. In one case, they could identify a causative factor: the patient ceased the topical 1% Cyclosporine A treatment. Apart from the clinical features of immune rejection (epithelial edema, epithelial rejection lines, persistent peripheral epithelial defects in six eyes, aggravation of vascularization in eight eyes and stromal opacity in nine eyes), impression cytology was performed in six cases and it revealed CD4^+^ and CD8^+^ cells. *In vivo* confocal microscopy demonstrated the presence of network-like Langerhans cells in central and peripheral cornea. All eyes were however restored to a stable ocular surface with anti rejection treatment.

The corneal surface following allogenic CLET was analyzed by Chen et al. (30), who included 41 eyes of 41 patients. The mean follow-up time was 22.13 months. Clinical success was recorded in 32/41 eyes (78%). Authors analyzed by immunohistochemical staining 41 pannus specimens and found positive staining for K19 in all specimens. Sporadic staining for K3 and P63 was also recorded. Histology also revealed a higher number of cell layers compared to normal corneal epithelium. They also performed impression cytology and subsequent PCR and STR analysis for 14 pannus specimens and 5 corneal buttons in order to establish if DNA donor was present after 3 months. All specimens were negative for DNA donor. Therefore, speculation that the multilayered epithelium could be residual cultured cells was invalidated. Authors speculated that transplanted cultured cells could be affected by a chronic immune rejection and accumulation of inflammatory cytokines. They also speculated that a residual number of residual stem cells of the host are stimulated to proliferate, therefore ensuring stability to the ocular surface.

Wang et al. (15) compared results of allogenic CLET to COMET. Seventy-six eyes of 73 patients were included: 41 patients (42 eyes) in the allogenic CLET group and 32 patients (34 eyes) in the COMET group. Mean follow-up was 23.3 months for allogenic CLET and 16.1 months for COMET. A higher success rate was recorded in the allogenic CLET group compared to COMET (71.4%, respectively, 52.9%, *p* = 0.043). Authors found higher failure rate that was associated with eyelid abnormalities and following COMET (3.5 times higher than allogenic CLET). The complications following allogenic CLET were PED (three cases), recurrent symblepharon (four cases), recurrent corneal conjunctivalization (four cases), and immune rejection (one case). In the COMET group, nine had PED, four had recurrent symblepharon, and three cases had recurrent corneal conjunctivalization. In allogenic CLET, 47.6% showed improved vision by one line ore more (compared to 50% in COMET), while two eyes (4.8%) worsened (compared to COMET, three eyes worsened, 8.8%) and the rest showed no improvement. There was no statistically significant difference in preoperative and post-operative vision between the two groups. Although allogenic CLET poses a rejection risk, anti rejection therapy was effective, and graft failure due to rejection was rare. The authors suggested that, because patients treated with COMET were more likely to develop persistent epithelial defects, leading to graft failure, allogenic CLET should be prioritized.

### COMET/CAOMECS Studies

Nishida et al. (35) reported in 2004 complete re-epithelization at 1 week following COMET and the central corneal surface maintained its transparency during the 14 months follow-up period. On the other hand, the rate of success was not mentioned in the study and authors did not report any complications.

Nakamura et al. (37) evaluated clinical results (conjunctivalization, corneal opacification, neovascularization and symblepharon formation) following COMET according to their grading system. Improvement of more than one line of visual acuity was documented in 53% of cases at the 36 months follow-up. Out of 19 eyes which received COMET, 7 eyes (37%) were documented with at least one episode of persistent epithelial defect during the follow-up period and three eyes (16%) presented ocular hypertension.

The main outcomes of Satake et al. (38) were a transparent corneal surface, without fibrovascular invasion and a functional fornix. Improvement of visual acuity was also recorded but additional surgery (keratoplasty) was required for better results than COMET alone. Complications like fibrovascular invasion were found in 20% of cases and persistent epithelial defects were present in 22.5% of patients, with a higher rate among limbal stem cell deficiency following chemical or thermal burns and Stevens-Johnson syndrome.

Following CAOMECS, a success rate of 75% was reported by Burillon et al. (13) during the 12 months follow-up period. Patients also had an improvement as far as photophobia, pain and visual acuity were concerned. Two patients out of the 25 included in the study were classified as graft failures. Sotonozo et al. (40) mainly focused on the improvement of visual acuity following COMET and concluded that this was influenced by factors like: preoperative visual acuity, etiology of LSCD, age of patient, two-step surgery, concomitant cataract surgery or transplant of amniotic membrane. Visual prognosis was better in cases of severe neovascularization. They described the rate of success regarding visual acuity according to the etiology. They included 40 patients in their study, 17 cases of Stevens-Johnson syndrome, nine patients with ocular cicatricial pemphigoid, 6 cases of chemical/thermal injury and eight patients with other causes of limbal stem cell deficiency. They recorded an improvement in 50% of cases suffering from Stevens-Johnson Syndrome, 42.9% for ocular cicatricial pemphigoid and 20% for LSCD following burns. However, authors mentioned that 48.9% of the patients included in the study had previously unsuccessful corneal or amniotic membrane transplantation. In 40% of cases there was a persistent epithelial defect following COMET and 5% of patients presented with corneal melting, however, no perforation was recorded.

Kolli et al. (41) followed two patients after COMET and in both cases they documented a stable epithelium, improvement in visual acuity and regarding ocular pain and discomfort.

Kocaba et al. (14) studied the long term results of CAOMECS in bilateral LSCD and documented an improvement regarding visual acuity in 74% of cases. Furthermore, the 23 patients included in their study were divided into two groups: one which underwent corneal transplantation and one which did not. Mean follow-up duration was 28 months. There was no significant difference between the two groups regarding further visual acuity improvement. The authors did not mention the interval between CAOMECS and penetrating keratoplasty. Two graft failures were reported in the group which underwent corneal transplantation.

Dobrowolski et al. (42) studied 13 cases of aniridia patients in which a stable corneal epithelium was observed during the observation period of 12–18 months. There were 3 cases of stromal scarring and vascularization and conjunctival vascularization. These cases were classified as graft failure.

Kim et al. (43) in a prospective clinical trial which studied the survival of COMET reported a rate of success of 75% and improvement in visual acuity in 62,5% of the cases at the 6 months follow-up. Their study included eight subjects, age ranging from 17 to 60 years. In four cases, penetrating keratoplasty was performed for residual stromal opacity following COMECs, after obtaining a stable corneal surface for a minimum of 6 months. Persistent epithelial defects were present in 50% of the cases at follow-up and were treated with amniotic membrane transplantation or semi scleral lens. No other adverse reactions were recorded.

Priya et al. (39) found a success rate lower than mentioned previous studies. The study included 10 male patients with bilateral chemical injuries or Stevens Johnson syndrome. Patients aged 8–65 years old were followed for a mean duration of 18.6 months. Anatomical success was recorded in 3/7 patients (42%) and visual improvement was present in 2/7 cases (28%).

## Discussions

**Figure 1:**
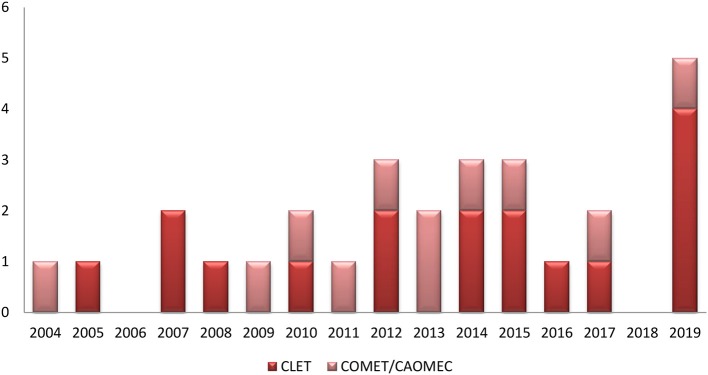
Distribution of clinical studies from 2004 to 2019; comparison between CLET and COMET/CAOMECS.

The time distribution of clinical studies for CLET and COMET shows a homogenous interest in both of the procedures during the past 15 years (Figure 1). However, the number of patients included was very low, with mostly small series ranging from 2 to 40 patients. One exception was the study of Cheng et al. (31), which followed 80 patients with symblepharon for more than 12 moths.

A comparison between CLET and COMET/CAOMECS, regarding the results, is not fully possible. The small number of patients is divided between various pathologies with very different outcome: Sjogren Syndrome, burns (chemical or physical), ocular cicatricial pemphigoid, persistent epithelial defects, or aniridia. Even in CLET the method of cultivation was not consistent, some authors using explants only, others suspension cultures and others using both of them (19). The success of the graft is further influenced by the quality of donor material. Both cadaver and living matched donors were used, with similar outcomes. The success of the graft depends on whether the number of cultivated cells is sufficient or not. During *ex vivo* culture, only some limbal epithelial progenitors migrate onto the amniotic membrane. Some of the cells undergo mesenchymal transition (48). There was no difference between the methods of cultivation reported. Both explants and suspension cultures showed similar results. Zakaria et al. (27) also showed the viability of a xeno-free cultivation technique.

To further complicate the evaluation, the graft success was quantified by different criteria, from DNA analysis (18), impression cytology (7, 18, 19, 24), confocal microscopy (28) to clinical evaluation (visual acuity improvement, lack of vascularization of the cornea or stable epithelium). Sometimes the visual acuity was not improved although the ocular surface showed signs of improvement. These cases usually underwent additional surgical procedures, for instance corneal transplants. COMET or allogenic CLET seem to improve the outcome of such additional procedures (37).

Corneal healing is a fascinating topic although poorly understood. There is no precise mechanism for the function of the grafted stem cells. It is possible that some residual stem cells in total LCSD still remain in dormant state and are stimulated by donor stem cells to multiply (18). Donor stem cells seem to vanish few months after transplantation. In 2005, Daya et al. (18) first noted that after about 9 months there were no more donor cells and no DNA from the donor to be found in the allogenic CLET area. That suggested that CLET has no clear need of autologous source of stem cells or a well-matched living relative, as the cells will be totally replaced months after the grafting. Non-matched cadaveric stem cells from discarded limbal rings could be an adequate source. This could explain the lack o difference between the clinical outcomes from allogenic CLET and COMET, both with more than 50% rate of clinical success. Different authors showed that cells covering the cornea retain a corneal phenotype. Basu et al. (23) found no goblet cells in the successful grafts and epithelial cells expressed specific K12 corneal marker.

Because of autologous origin and lack of immune rejection, mucosal stem cells are expected to perform better than allogenic stem cells. However, Wang et al. (15) showed that the morphology, function, and microenvironment of oral mucosal epithelium are very different from those of limbal epithelium, despite the expression of similar gene markers. They observed a higher incidence of persistent epithelial defects in COMEC than allogenic CLET.

As far as surgery success according to individual factors is concerned, it is difficult to draw conclusions from the studies mentioned in this review. Because of the heterogeneity of the data included in the studies, it is difficult to establish whether age, gender or cause of the limbal stem cell deficiency can influence the survival of the graft and success rate following surgery.

Shimazaki et al. (19) found a higher success rate in patients suffering from Stevens-Johnson syndrome, however they did not study individual factors. Satake et al. (38) also studied the rate of success according to the cause of LSCD. Patients with Stevens-Johnson syndrome had a more stable epithelium after CLET than patients with LSCD following burns or OCP. In one study which studied results of CLET in patients with aniridia, 3 out of the 13 female patients included had a lower rate of epithelium regularity (42). Ganger et al. (29) followed the long-term outcomes in children and adults following CLET, however comparison between the two groups was not done due to the small number of patients.

Following COMET, one study detailed results according to the disease. They concluded that patients with Stevens-Johnson syndrome had the greatest improvement in visual acuity. In patients with OCP and age over 60 years improvement in visual acuity was the best at 4 weeks post-operative but it later diminished (40).

One meta-analysis (49) also concluded that success rates are difficult to be compared due to the heterogeneity of the data, however, local factors, such as adnexal pathology, should be corrected for a better outcome.

Future perspectives for treating total bilateral limbal stem cell deficiency are currently under evaluation. Studies have shown promising results regarding using different cell sources. Hair follicle bulge-derived stem cells provided a good corneal surface after transplantation in murine models. Human immature dental pulp stem cells, embryonic stem cells and umbilical cord stem cells also showed good results in animal models (50, 51). One study (52) compared results between allogenic CLET and allogenic bone marrow-derived mesenchymal transplant (MSCT). They included 11 patients who underwent allogenic CLET and 17 who had MSCT. After a follow-up period of 6–12 months, they concluded that both surgical techniques had similar rates of success, 77.8% CLET, respectively, 85.7% MSCT.

Somatic cell reprogramming by using viral vectors was first evidenced by Chakrabarty et al. (53) resulting induced pluripotent stem cells (iPS). Even though iPSC might play a major role in regenerative medicine, there are a few concerns regarding their use. They carry an important risk of genomic instability which might influence their clinical applicability. Other issues related to their use are the potential tumorigenicity and the necessity of a longer cultivation period. The culture duration is dependent on the age of the donor, the phenotype of the reprogrammed somatic cell, and the cultivation conditions. The major advantage is the absence of immune rejection. The potential of iPS has started to be explored in human subjects. In Japan, Nishida described one of the first patients treated with iPS, with promising results (54).

## Conclusions

The aim of our review was to summarize clinical outcomes following allogenic CLET and COMEC/CAOMECS. The visual acuity improvement in both methods was reported similar, with percentages usually higher than 50% of the treated cases. However, the comparison between studies was difficult to achieve due to the lack of a universal and objective grading tool for assessing post-operative results. Moreover, some studies which reported results following both auto and allogenic CLET, described mixed results for both procedures, therefore analyzing the results separately was impossible. As a further matter, only one study compared preoperative and post-operative aspects of limbal stem cell deficiency using *in vivo* confocal microscopy and impression cytology.

The differences in complication rate were difficult to assess due the fact that studies evaluated differently their outcomes. It was difficult to describe the impact of individual factors, such as age, gender, or cause of LSCD on the surgical outcomes due to the lack of sufficient data. COMET/CAOMECS may present certain advantages compared to CLET. These advantages are represented by overcoming the problems associated with allograft rejection, the achievement of cell culture in a shorter period of time and the absence of keratinization during a prolonged time span. However, a study comparing allogenic CLET and COMEC suggested that oral mucosal epithelial cells have lower success, due to the higher incidence of post-operative PED and graft failure, and lower cell proliferation and differentiation activities. Allogenic LESCs may have a better ability to form a stable and integrated corneal epithelium.

## Author Contributions

OS and DG had the same contribution and should be considered first author. Database search was performed by DG and OS independently in order not to miss any relevant work in the researched field. The review was written and reviewed equally by both authors.

### Conflict of Interest

The authors declare that the research was conducted in the absence of any commercial or financial relationships that could be construed as a potential conflict of interest.
